# ‘I Am Not The Doctor For You’: Physicians’ Attitudes About Caring For People With Disabilities

**DOI:** 10.1377/hlthaff.2022.00475

**Published:** 2022-10

**Authors:** Tara Lagu, Carol Haywood, Kimberly Reimold, Christene DeJong, Robin Walker Sterling, Lisa I. Iezzoni

**Affiliations:** Northwestern University, Chicago, Illinois.; Northwestern University.; University of Massachusetts, Worcester, Massachusetts.; Baystate Health, Springfield, Massachusetts.; Northwestern University.; Harvard University and Massachusetts General Hospital, Boston, Massachusetts.

## Abstract

People with disabilities face barriers when attempting to gain access to health care settings. Using qualitative analysis of three physician focus groups, we identified physical, communication, knowledge, structural, and attitudinal barriers to care for people with disabilities. Physicians reported feeling overwhelmed by the demands of practicing medicine in general and the requirements of the Americans with Disabilities Act of 1990 specifically; in particular, they felt that they were inadequately reimbursed for accommodations. Some physicians reported that because of these concerns, they attempted to discharge people with disabilities from their practices. Increasing health care access for people with disabilities will require increasing the accessibility of space and the availability of proper equipment, improving the education of clinicians about the care of people with disabilities, and removing structural barriers in the health care delivery system. Our findings also suggest that physicians’ bias and general reluctance to care for people with disabilities play a role in perpetuating the health care disparities they experience.

More than sixty-one million Americans had a disability as of 2016.^1^ Disparities in health care access and quality have been observed across many groups of people with disabilities and in a variety of clinical environments.^2–5^ People with disabilities also have been found to be less likely to report satisfaction with their care compared to people without disabilities.^6–8^ Disparities in access to health care and the quality of that care have been associated with worse physical health and greater burden of chronic disease for people with disabilities compared to their nondisabled peers.^9,10^

Disparities in health care persist despite the Americans with Disabilities Act (ADA) of 1990 and the ADA Amendments Act of 2008, which mandate equal access to health care services. Multiple factors contribute to these disparities: physical inaccessibility of care settings;^11,12^ inadequate accommodations for communication needs;^6,7^ and pervasive ableism in medicine,^13–15^ including physicians’ implicit and explicit biases, attitudes, and behavior toward people with disabilities.^16–18^ However, physicians’ attitudes about caring for patients with disabilities in community settings rarely have been explored.^19,20^ As part of a larger mixed-methods study, we therefore aimed to further explore community primary care physicians’ and specialist physicians’ perspectives on caring for people with disabilities.

## Study Data And Methods

### PARTICIPANT RECRUITMENT

We conducted three videoconference focus groups in October and November 2018: one with non-rural-practicing primary care physicians, one with physicians from selected specialties (rheumatology, neurology, obstetrics/gynecology, orthopedics, and ophthalmology), and one with rural-practicing primary care physicians from across the US. We used a professional social networking site for physicians (Sermo) to recruit eight to ten physicians for each focus group.^21^ At the time of this research, Sermo had approximately 800,000 verified physicians from more than 150 countries across ninety specialties.^22^ Sermo identified participants following our recruitment criteria, which included physicians’ specialty, gender, race and ethnicity, urban or rural location, non-trainee status, and active outpatient practice in the US with at least some patients with selected disabilities. Sermo arranged $200 incentive payments and shared only participants’ basic demographic information and state but no identifying information. We did not receive any information about physicians who were screened but did not participate in the study. The conduct of focus groups was approved by the Massachusetts General Hospital Institutional Review Board (IRB); the qualitative analysis portion of this study was deemed to not be human subjects research by the Baystate Health IRB because transcripts were deidentified.

### INTERVIEW GUIDE DEVELOPMENT

We designed and piloted a semistructured interview guide based on literature reviews and prior studies of health care and people with disabilities.^13,23,24^ The interview guide was organized to discuss issues relating to care for people with specific disability types (mobility, hearing, vision, mental health, and intellectual disabilities). The full interview guide is in the online appendix.^25^ We also asked about knowledge and application of the ADA and general barriers to and facilitators of caring for people with disabilities.

### FOCUS-GROUP PROCEDURES

Sermo contracts with a web-based video platform to support online interactions. During the focus groups, real-time video streams of all participants, including the moderator, appeared on the screen simultaneously, along with participants’ first names or nicknames. The multiuser display allowed the moderator (Lisa Iezzoni) to identify visual cues when participants wanted to speak and revealed other nonverbal information (for example, head nods and facial expressions).

Each focus group lasted approximately two hours. The moderator began by introducing herself as a researcher developing a national survey on physicians’ experiences with and views of caring for adults with functional impairments. Participants were told that the group members shared a specific characteristic (for example, all primary care physicians or all specialists). Participants and the moderator were visible during the entire focus-group session. Other members of the research team observed the focus groups off screen (that is, not visible to participants); participants were informed of their presence. These observers occasionally communicated privately with the moderator (for example, suggesting follow-up questions) through the site’s chat feature.

The focus groups were recorded and transcribed for analysis. One team member reviewed associated videos and added contextual notes (for example, number of hands raised, nods, and silence) to the transcripts.

### ANALYSIS

We uploaded transcripts into QRS NVivo 12 Pro qualitative data management and analysis software. We applied the constant comparison method of coding,^26^ using inductive and deductive analysis to build the coding structure from prior work and an initial review of the transcripts. To refine the initial coding scheme, two members of the research team drafted analytic memos and co-coded one transcript. Transcripts were also reviewed separately by the moderator and other research team members for theme identification. Before coding individually, coders compared analytic memos with one another, discussed discrepancies, and expanded the codebook to capture focus-group dynamics until agreeing on a revised codebook. To verify the analysis, the research team met to review and discuss the identified themes; on completion, they reached consensus about thematic saturation.^27^

### LIMITATIONS

This study had limitations. A commercial organization (Sermo) drew an anonymous convenience sample from an online community, which might not represent US physicians generally. Because we received very little information from Sermo about participants, we were unable to compare participants with US physicians or Sermo members. Further, although Sermo did not tell possible participants that the study was about disability, people who responded and participated in the focus groups may have been different than those within the Sermo community who did not respond. The sample also included very few academic physicians. However, the sampling method allowed us to identify a diverse group of community physicians who practiced in a variety of fields with broad geographic distribution. The anonymity may have created a feeling of safety that allowed physicians to speak with candor.

The interview guide was designed to be broad for purposes of hypothesis generation, but this limited our ability to ask more specific questions in this study, including the role of physician geography and rurality; details about access issues specific to communication, physical, and intellectual disabilities; and details about the processes of care that are most in need of urgent improvement. Although the breadth of the study was a limitation, it also allowed us to identify many possible opportunities for future work to explore.

In cases where care refusals were described, we were not able to clarify whether the physician was describing the use of legitimate reasons to turn away patients (for example, the physician actually did not take the patient’s insurance or was not accepting new patients) or describing excuses that were not true. Finally, we tried to determine whether there were differences in themes between primary care and specialist physicians but were unable to do so.

## Study Results

### PHYSICIAN AND PRACTICE CHARACTERISTICS

The focus groups included a total of twenty-two participants (exhibit 1). Mean age was 51.3 years; fourteen participants identified as male and ten as White. Fourteen participants were primary care physicians, seven of whom practiced in rural regions, and eight were specialists. Fifteen participants described their practice as small (one or two physicians) (data not shown).

Exhibit 2 presents the overarching themes in the areas of barriers to providing care to people with disabilities and physicians’ assessment of their knowledge of the ADA and their responsibilities under the law.

### BARRIERS TO CARING FOR PEOPLE WITH DISABILITIES

Focus-group responses identified several barriers to providing care for people with disabilities: physical accommodations; communication accommodations; knowledge, experience, and skills; structural barriers; and attitudes toward people with disabilities (with a subtheme describing discharging people with disabilities from their practices or denying care to them). Themes were intersecting, overlapping, and multidimensional. We previously published findings relating to two subthemes (not described here): physicians’ attitudes toward people with obesity^20^ and reproductive health access for people with intellectual disability.^28^

#### ▸ PHYSICAL ACCOMMODATIONS:

All participants reported physical barriers to providing health care for people with disabilities, including inaccessible buildings and equipment. Many participants were forthcoming about the lack of accessibility in their clinics. For example, one rural-practicing primary care physician said, “I know for a fact our building is not accessible.” When asked about access to automatic height-adjustable exam tables, some described these tables as an asset, but others seemed more ambivalent. As a non-rural-practicing primary care physician said, adjustable-height exam tables are “designed to be adjustable for the practitioner, not for the patient’s comfort or the patient’s ability to get in. …They are kind of clunky.” Access to transfer equipment (for example, a Hoyer lift) or accessible weight scales was rare across the groups. Some participants reported using workarounds for physical accommodations, such as low exam tables. In response to the question, “If a wheelchair user comes and cannot stand on a weight scale, what is your approach to taking a weight?,” physicians from two of the three groups reported sending patients to a supermarket, grain elevator, zoo, or cattle processing plant to obtain a weight. More details about their responses to this question have previously been reported.^20^

#### ▸ COMMUNICATION ACCOMMODATIONS:

Participants discussed various approaches to communicating with people with vision or hearing impairments and those with intellectual disabilities or mental illness as part of clinical care. None of the participants was able to provide patients with written materials in Braille, and only a few offered print materials in large type. Physicians across the three groups reported relying frequently on caregivers or written communication to overcome barriers. In response to the question, “Do you have approaches for ensuring you are communicating effectively with patients with intellectual disability or serious mental illness?,” one primary care physician stated, “I’m fortunate that my patients who use sign language usually bring someone with them. …But also, we use pen, paper, and a whiteboard.” Referring to patients with hearing loss, another primary care physician said, “A lot of times, the caregivers are able to give us a lot more information without communicating with the patient directly. So that’s how we get the information that we need: from the caregivers.” Caregivers were identified as an essential tool for health care encounters. We did not directly inquire about whether the physicians asked patients about communication preferences, but few participants asserted that they talked to the patient, regardless of the patient’s known ability to communicate. Additional details on the focus-group participants’ and other physicians’ attitudes related to communicating with patients with intellectual disability have been published elsewhere.^28^

Participants described both financial and time-related challenges of accommodating communication needs. One non-rural-practicing primary care physician stated: “I took it upon myself to actually hire an outside service to do [sign language interpretation]. They billed the office. …Their bill was higher than what we were making, so it was a losing venture. …It cost me $30 per visit for that patient, out of pocket.”

Many physicians expressed explicit bias toward people with disabilities.

Physicians described providing virtual interpreting services for patients (for example, via iPad), but nearly all reported that any additional technology aids patients needed (for example, augmentative and alternative communication devices and screen readers) were provided by the patients themselves.

#### ▸ KNOWLEDGE, EXPERIENCE, AND SKILLS:

Physicians in each of the three groups noted the lack of sufficient knowledge, experience, and skills among themselves and clinic staff concerning care for people with disabilities. Patient transfer skills were mentioned often—specifically, a fear of hurting themselves or their patients. For example, one specialist physician said, “If I am trying to transfer the patient and they fall and hurt themselves, I am not sure what I accomplished.”

Most participants did not express clear feelings of obligation to provide accommodations when patients came with their own support. As one primary care physician said, “I haven’t had a lot of experience with [patients who are] hearing impaired. Typically, they come with caregivers.”

#### ▸ STRUCTURAL BARRIERS:

Physicians in all three groups discussed structural barriers to providing care for people with disabilities, which we coded into three categories: procedural, policy, and financial or allocation of resources. Subthemes included lack of time with patients, burden of documentation and paperwork, difficulties with coordination of care, lack of awareness that a patient requiring accommodations is scheduled, and lack of communication about the needs of people with disabilities.

Physicians across groups described ways in which structural barriers limited their ability to provide care that aligned with patients’ and families’ needs. Participants repeatedly raised the issue of limited time with patients as a barrier to providing high-quality care to people with disabilities. One participant, a non-rural-practicing primary care physician, called current appointment lengths “unreasonable” and “unacceptable.” A rural-practicing primary care physician said, “It’s hard to individualize what you need to do and make sure they understand, and you take care of their needs, in a fifteen-minute appointment.” Physicians described time constraints affecting their ability to “get through the day,” with one specialist saying that people with disabilities were “a disruption to clinic flow.”

Time constraints impeded physicians’ ability to coordinate care with families of people with disabilities, particularly when family members were not local or were unable to attend appointments. As a rural-practicing primary care physician stated, “I have found that with my geriatric patients, a lot of their family don’t live within that community, so coordination of care becomes a huge challenge and barrier.” Physicians frequently stated that their clinical settings failed to provide adequate expertise or administrative support needed to care for people with disabilities.

Physicians also raised concerns about scheduling and the ability to document the need for accommodations in the electronic health record. One specialist reflected: “I would love to say we are more system-organized, but I doubt there is any know-ahead that anybody with a disability is coming. When they get there, we make do and try to accommodate as best as we can, but it would [be] a surprise to me if I knew they were coming, and I don’t think the office manager knows, either.”

Participants also discussed limitations of the electronic health record in documenting accommodation needs from visit to visit. One primary care physician said, “We do have a place in the [electronic health record] that allows us to document what accommodations patients may need, but it’s basically a small sticky note on the side. …You could bypass one of them.”

#### ▸ ATTITUDES TOWARD PEOPLE WITH DISABILITIES:

Some participants across the three groups revealed negative attitudes about people with disabilities and commonly used outdated or ableist language (for example, “mentally retarded”). Many participants implied that providing accommodations to care for people with disabilities was burdensome. One specialist said about people with disabilities, “they can create a big thing out of nothing.” Another said that people with disabilities “are an entitled population.”

Multiple participants indicated that people with disabilities make up a small portion of their caseloads. A participant in the non-rural-practicing primary care group said, “You’re only going to have a certain percentage of patients that are going to require [accommodations]—maybe 10 percent, 15 percent—so how much can you do?” Similar comments were repeated across groups, suggesting limited recognition and de-prioritization of people with disabilities. When asked to describe what would lead physicians to purchase accessible equipment, for example, one primary care physician reported that their practice saw few patients with disability and thus had little need for accessible equipment: “If we had a practice that had even a 20 percent population [of people with disabilities], and I’m talking mental health or even physical disability and things like that, I think we can make a strong argument for some of these [accommodations]. The problem with that is, we already know there are tons of barriers to access for these patients to come in to begin with, so fewer of them come in than probably need to come in, and because very few come in, so it’s hard to make the argument to bring these things to bear for one or two patients.”

#### ▸ DENYING OR DISCHARGING PATIENTS:

Some participants described denying care to people with disabilities or attempting to discharge people with disabilities from their practices; these refusals were varied in their rationale. Some physicians described care that they would have provided if a patient did not have a disability. “We have had patients where the level of disability is too high, and it is such a very delicate procedure and delicate part of the human anatomy, and we felt we couldn’t control the situation enough to do it properly,” one specialist said. Another participant, a primary care physician, offered a clinical reason that the people with disabilities did not need care: “We talk to the caregiver or the patient or whatever and just explain that it is very unlikely that they’re going to develop cervical cancer.” In other cases, a specialist reported telling a patient that they needed more care than the practice could provide: “I think you need a lot more care, and I am not the doctor for you.”

Some physicians described their thought processes in these situations, sometimes acknowledging that they were aware of requirements that prevented them from denying care because of disability. As one specialist put it, “I think the problem is that you cannot refuse them straight. We have to give them an appointment. You have to come up with a solution that this is a small facility, we are not doing justice to you, it is better you would be taken care of in a special facility.”

In other cases, some physicians reflected on strategies that would allow them to discharge the patients but minimize risk for lawsuits or other consequences. “It can be turned around that a particular doctor’s office did not offer all the resources. I have actually thought about it a lot because in a sense we are kind [of] in a powerless position to deny care. …My solution is to say, ‘I no longer take new patients,’” one specialist said.

Improvements in medical education and training are needed to better prepare physicians to care for people with disabilities.

At least one specialist physician stated that they believed that failure to provide any care is nearly always the wrong clinical decision: “I would be hard pressed to think of a situation where no care rendered by the physician is worse than some care being partially rendered.” And a primary care physician said, “I’m not sure I could come up with a scenario where I could say I would refuse someone with a disability.”

### KNOWLEDGE OF THE ADA

When asked about their knowledge of the ADA, nearly all of the physicians reported having little or no training on the law and its implications for their practices. A participant who is a specialist said, “I know they offer conferences and lectures, but this is a personal choice if you want to take it or not.” In general, attitudes about the ADA were apathetic and even adversarial. For example, one specialist physician described feeling as if the legislation works “against physicians” and thereby does not help people with disabilities.

## Discussion

Across focus groups, community-based primary care and specialist physicians in urban and rural settings identified a broad range of barriers to caring for people with disabilities. Many physicians also expressed explicit bias toward people with disabilities and described strategies for discharging them from their practices. Physicians raised concerns about the expense of providing physical and communication accommodations, including insufficient reimbursement for physicians’ efforts and competing demands for staff time and other practice resources. Many participants described caring for very few patients who need accommodations, with little acknowledgment that the barriers to obtaining care and inability to track or respond to accommodation needs could lead to an underidentification of the number of people with disabilities who seek care. This study adds to the understanding of attitudes among community-based physicians toward people with disabilities and provides context to recent survey findings that physicians frequently do not welcome them into their practices.^13,18^

Perpetuation of inequitable care for people with disabilities is inconsistent with the mission of medicine and public health.

Previously, we reported the results of a survey of 714 practicing US physicians (primary care physicians and subspecialists).^16–18^ We found that only 41 percent of respondents reported that they were “very confident” about their ability to provide the same quality of care to people with disabilities as those without, and just 57 percent strongly agreed that they welcomed people with disabilities into their practices.^16^ Most physicians reported that they do not use accessible equipment for routine care of patients with chronic, significant mobility limitations; fewer than one-quarter attempted to regularly weigh people with disabilities; and only 40 percent always or usually used accessible exam tables or chairs.^18^ There were clear gaps in knowledge about requirements of the ADA: 36 percent reported knowing “little or nothing” about their legal responsibilities under the ADA, and nearly 70 percent reported that they were at risk for ADA-related lawsuits.^17^ Taken together, the focus groups and survey responses provide a substantive and deeply concerning picture of physicians’ attitudes and behaviors relating to care for people with disabilities.

Prior research also suggests that physicians feel burdened by time constraints in their practices, even when accommodations are not involved.^29,30^ As we report in the results, some of the participants reported that their practice settings do not provide sufficient administrative or clinical support for the care of people with disabilities. Our physician survey found that 13.6 percent and 31.1 percent of participants, respectively, felt that time constraints were a large or moderate barrier to caring for people with disabilities.^17^

The stated goal of the ADA is “to assure equality of opportunity, full participation, independent living, and economic self-sufficiency for” people with disabilities in light of “the continuing existence of unfair and unnecessary discrimination and prejudice” against people with disabilities in our society.^31^ It is patterned after Section 504 of the Rehabilitation Act of 1973, which prohibits discrimination against people with disabilities in federally funded programs and services. As a piece of civil rights legislation, the ADA includes both public-sector services (Title II) and private services available to the public (Title III) and is not discretionary. Physicians cannot legally discriminate against a patient because of disability.

However, it is difficult to enforce the ADA in medical settings. Discretion is part of physicians’ treatment decisions, and clinical decisions that occur in real time can often be justified. The explanations physicians gave in this study could, for any single case of denying care, be legitimate (for example, not accepting the patient’s insurance or denying the patient’s need for a desired clinical service). Although disparities in care for people with disabilities suggest that there is a pattern of more frequently denying care to them than to people without disability,^11,32^ it is nearly impossible to know whether any single situation was discrimination related to disability.

The ADA is also difficult to enforce because it depends on people with disabilities presenting discrimination concerns to the Department of Justice. The burden is on the person with a disability to file a complaint or lawsuit.^33^ Additionally, the excuses provided by the physicians in this study seem plausible; it would, therefore, be nearly impossible to establish that the physicians intended to discriminate against patients with disabilities.

## Implications

This study and prior work suggest that people with disabilities are frequently not accommodated in health care settings, often receive substandard care, and in some cases are refused care. There is an urgent need to better understand and address clinician- and system-level barriers to care for people with disabilities. Further large studies of system-level interventions are needed as well. For example, mandated documentation of disability status and accommodation needs in the electronic health record could help clinicians and practices prepare for the needs of people with disabilities ahead of a planned visit.^34^ Improvements in medical education and training are needed to better prepare physicians to care for people with disabilities. The range of barriers and negative attitudes expressed by participants in our study, however, suggest that improving the accessibility of health care settings and establishing disability education standards for clinicians are both necessary but are not sufficient to ensure equal quality and accessibility of care for people with disabilities.

Physicians’ biases and discriminatory attitudes appear to play a significant role in perpetuating health disparities for people with disabilities. Physicians, administrators, and policy makers must continue to use all available tools (education, publicity, lawsuits, and policy levers) to address the negative consequences of the stigmatizing attitudes expressed by physicians in this study. Perpetuation of inequitable care for people with disabilities is inconsistent with the mission of medicine and public health.

## Supplementary Material

Appendix

## Figures and Tables

**EXHIBIT 1:** Characteristics of participants in physician focus groups discussing caring for people with disabilities, fall 2018

Characteristics	
Mean age, years (SD)	51.3 (9.9)
Age range, years	35–67
Gender, no.	
Male	14
Female	8
Race, no.	
White	10
Other^a^	12
Hispanic ethnicity	2
Primary care (rural), no.	
General internal medicine	2
Family practice	5
Primary care (nonrural), no.	
General internal medicine	4
Family practice	3
Specialty (nonrural), no.	
Rheumatology	2
Neurology	2
Obstetrics/gynecology	2
Orthopedics	1
Ophthalmology	1
Time in practice, years	
5–10	6
11–20	9
21–30	6
31+	1
Type of practice, no.	
Private, not hospital-based	19
Hospital-based practice	2
Other	1
US region of practice, no.	
South	8
Midwest	8
West	3
Northeast	3

**SOURCE** Participant demographic questionnaire administered by Sermo. **NOTE** *N* = 22.

a For focus-group recruitment, Sermo allowed participants to self-identify race. Of those who designated “other,” five identified as Asian/Pacific Islander, one as African American Black Caribbean, one as Jewish, one as Indo-Pakistani, and two as mixed (with one participant specifying Asian and Caucasian).

**Exhibit 2:** Analytic themes illustrating barriers to caring for people with disabilities, with selected responses, from physician focus groups, fall 2018

**Themes and selected responses**
**PHYSICAL ACCOMMODATIONS**
We have issues with power chair or wheelchair patients who couldn’t come in the front door; we had to make a ramp in the back entrance of the clinic so they come through the back door.
I think our [medical assistants] just put wheelchair “w/c” and the weights don’t get checked until someone makes a big deal about it, and the argument is same: “it’s not safe.”
**COMMUNICATION ACCOMMODATIONS**
I use paper and pen. And most of my patients have hearing aids that are not working... It’s just better to use paper and pen, sometimes it’s just better, because with HIPAA, when they’re yelling and you are yelling, the whole office can hear you yelling.
**KNOWLEDGE, EXPERIENCE, AND SKILLS**
Durable medical equipment, that’s a very big barrier. And not even knowing myself what would be the best kind of care, the best equipment for them, I don’t even know, I’m not even qualified.
**STRUCTURAL BARRIERS**
Seeing patients at a 15-minute clip is absolutely ridiculous. To have someone say, well we’re still going to see those patients with mild to moderate disability in those timeframes—it’s just unreasonable and it’s unacceptable to me. But training [to address problems common for people with mild to moderate disability] would help.
I have, like, 18 pages of [disability] documentation—of which 1 paragraph is essential and necessary for me to care for the patient.
Coordination of care becomes a huge challenge and barrier. Our institution is trying to get social workers in our office to do some of this legwork. There’s financial and space constraints that limit that, too, but we’re looking for solutions to be able help coordinate for care these patients with special needs because they are a unique population that require a unique set of interventions.
**ATTITUDES TOWARD PEOPLE WITH DISABILITIES**
We’ve gotten to a point in society where a lot of people are wanting some form of accommodation and a lot are illegitimate. They want their pet peacock on the airplanes and whatnot, and it makes it very difficult.
We remind the residents that the relationship is with the patient, and the guardian is there facilitating that relationship. But we kind of wanted to keep them focused on the fact that you are dealing with a living breathing human, regardless of the fact that you are communicating everything that needs to be communicated.
**KNOWLEDGE OF THE ADA**
I truthfully think the [Americans with Disabilities] Act makes the disabled person more of a target and doesn’t help them but hurts them. Because a lot of us, me personally, are afraid to treat them...so I look at it as not [a] helpful act, but I look at it as a hurtful act. Because all of us, even in this discussion, well, we are afraid of this, we’re afraid of that. ... You just don’t want to deal with them, and that’s what the [ADA] is all about.
I think we’re pretty open as sitting ducks for lawsuits if we try to get rid of a patient with disabilities because they can turn around and say that it was discrimination.

**SOURCE** Authors’ analysis of focus-group discussions. **NOTE** HIPAA is Health Insurance Portability and Accountability Act.
