# Supplementary material for: ‘I Am Not The Doctor For You’: Physicians’ Attitudes About Caring For People With Disabilities
Source: Health Aff (Millwood). Author manuscript; Available in PMC 2023 Mar 3. (PMC9984238; doi:10.1377/hlthaff.2022.00475)
Supplement: Appendix [file NIHMS1867640-supplement-Appendix.docx]

**Appendix 1: Focus Group Moderator’s Guide**

*Following introduction to study, moderator, and research team:*

1. I want to start by going around and having you introduce yourself by your first name, and then tell us your specialty and very briefly – only a minute – about whether your patient panel includes adults with difficulties with mobility, seeing, hearing, speaking, or who have serious mental illness or intellectual disability – and which of these conditions are most common in your panel.
2. What if you had a patient come for a visit, who is a long-time wheelchair user and cannot independently transfer – Would you examine that patient in their wheelchair? If not, how would that patient get onto an exam table?

PROBES:

- How would you make that decision about whether to examine the patient in their wheelchair?
- Do you have an automatic, height adjustable exam table?
  - If so, what do you think of that height adjustable exam table?
- Do you have a lift device, like a Hoyer lift?
  - If so, what do you think of that lift device?
- How do your staff assist patients with mobility impairments in the exam room?
- Have your staff ever been injured transferring patients?

1. The same patient cannot stand on a scale to be weighed - How would you think about getting a weight for this patient?

PROBES:

- Do you get weights on all your patients routinely?
  - If so, what would your thinking be about getting a weight for this long-term wheelchair user?
- Do you have a wheelchair-accessible weight scale in your office?
  - If so, what do you think about that weight scale?

1. What if someone who is deaf or hard of hearing comes to visit you - How would you approach communicating with that patient?

PROBES:

- Sign language interpreters? Who would organize that?
- In-person sign language interpreters or remote access?
- CART reporters?
- Assistive listening devices?
- Closed captioning on TV in waiting room?

1. What if someone who is blind or has low vision comes to visit you - If you give out written material, how do you approach making sure they can read it?

PROBES:

- Braille?
- Large font?
- Allowing people to audio-record?
- Web materials accessible using screen readers?

1. Do you have approaches for ensuring you are communicating effectively with patients with intellectual disability or with serious mental illness? I am combining these very different groups simply in the interest of time.

PROBES:

- Approaches toward ensuring communication, such as drawing, using dolls or models?
- Soothing environments, such as adjusting lighting or noise?

1. What are the major barriers that you face when caring for patients with functional impairments, like the ones we have been talking about?

PROBES:

- Costs (e.g., capital equipment purchases), hourly fees (e.g., sign language interpreters, CART reporters)?
- Space?
- Staffing levels?
- Problems getting in and out of and around the building, including parking, restrooms?
- Training?
- Time involved for making accommodation?
- Reimbursement level for visit?

1. I’d like to talk now about what you know about the Americans with Disabilities Act (“the ADA”).

PROBES:

- Have you ever been taught about your responsibilities under the ADA?
- Are there circumstances where you can refuse to see a patient with a disability?
- Have you heard about disability accommodations? What does that phrase mean to you?
- Who makes decisions about what accommodations a patient with disability needs?
- Can you ask patients to make their own accommodations (e.g., bring in someone to help move them or do sign language interpretation)?
- Can you charge the person with a disability for their accommodation?
- Would separate facilities specializing in caring for patients with disability comply with ADA requirements?
- Are you worried about legal exposure relating to accommodating your patients?

1. What would make it easier for you to care for your patients with disability?
2. Is there anything we have not talked about today about caring for patients with disability that you feel it is important for us to know?

*[THANK YOU and closing.]*
